# Assessment of nasal base morphology using new proportion indices in Chinese

**DOI:** 10.1186/s40064-016-2997-4

**Published:** 2016-08-08

**Authors:** Zhenyu Yang, Xiaoyan Tan, Jun Fang

**Affiliations:** Hangzhou Plastic Surgery Hospital, 168 Shangtang Road, 4 District of Zhaohui, Hangzhou, 310014 Zhejiang People’s Republic of China

**Keywords:** Nasal base, Proportion indices, Morphology, Han Chinese

## Abstract

**Objective:**

The aim of this study was to measure the soft tissue of the external nasal base in Han Chinese women and identify indices with which to simplify morphological assessment.

**Methods:**

This study involved 155 Han women in China. The control group comprised 101 women, and the surgical group comprised 54 women. Using measurements and analytical software, we measured the nasal base on photographs in the two groups. The nasal base was also measured preoperatively and postoperatively in the surgical group. Results from the two groups were compared with a *t* test.

**Results:**

The proportion index of the nasal tip triangle (upper nasal base) was c-prn/a = 0.33 ± 0.05 ≈ 1:3. The proportion index of the nostril trapezoidal (lower nasal base) was c-sn/all-alr = 0.25 ± 0.04 ≈ 1:4. The proportion indices of the nasal tip triangle and the nostril trapezoidal were larger in the postoperative surgical group than in the control group. Therefore, the nasal base morphology became stereoscopic through surgical correction.

**Conclusion:**

The current study provides a credible and objective reference for cosmetic nasal surgery. The proportion indices related to the nasal base can be used to intuitively and vividly ascertain the nasal base morphology.

*****Level of evidence***:**

III.

## Background

The nose is a prominent part of the face and affects an individual’s physical appearance. Different races and geographical regions are associated with specific external nasal characteristics, and different clinical reference standards should therefore be used. Anthropometric studies of different ethnic groups in recent years have found racial differences in the size, shape, and proportion of the nose.

Many studies on the nasal morphology of Caucasians, Asians, and Negroids have been performed for long periods (He et al. 2009; Wang et al. 2009; Ofodile and Bokhari 1995; Ofodile et al. 1993; Aung et al. 2000; Farkas 1994; Farkas et al. 1986; Li et al. 2014). Measurement techniques used in such studies include body measurement, computer-assisted digital photographic measurement, and three-dimensional laser scanning (He et al. 2009; Aung et al. 2000; Li et al. 2014). These techniques are used to obtain line and angle measurements and calculate the proportion index (He et al. 2009; Wang et al. 2009; Aung et al. 2000; Farkas 1994; Li et al. 2014; Sim et al. 2000; Daniel 2001; Mishima et al. 2002; Guyuron et al. 2005). All such research enriches the available data on the nasal morphology of different races and ethnicities.

Anthropometric measurements could help surgeons to perform objective and quantitative evaluations of deformities, make preoperative and postoperative assessments, and decide on surgical strategies (He et al. 2009). Simpler anthropometric measurements for preoperative nasal evaluation and analysis of postoperative changes are convenient in guiding rhinoplastic surgeons and in routine clinical application (Wang et al. 2009).

Nasal subunit plasty is currently a procedure of interest in the field of plastic surgery. A detailed analysis of nasal subunits based on intrinsic contour configurations was performed by Burget and Menick (1985, 1986). The surface of the nose is crossed by shallow ridges and valleys that separate it into slightly convex or slightly concave surfaces: the tip, dorsum, paired sidewalls, alar lobules, and soft triangles. These smaller parts (tip, dorsum, sidewalls, alar lobules, and soft triangles) may be termed topographic subunits.

Morphological research to date has not adequately addressed the nasal subunit, including the tip and the alar lobules. Aesthetic evaluation of the nasal base has also lacked objective and quantitative indicators. Few studies on the actual dimensions of the Han Chinese population have been performed (Ofodile and Bokhari 1995; Ofodile et al. 1993). It is important to establish reference values of the Chinese nasal base morphology.

In this study, 155 Han women in Zhejiang Province, PR China underwent measurement of the nasal base with analysis by computer-assisted measurement and analytical software. The obtained data provide a reference for soft tissue evaluation of the external nasal base.

## Methods

### Grouping

This study included 155 Han women in Zhejiang Province, PR China. We selected 101 women from the general population as the control group. Women in the control group had normal, harmonious facial features with no history of head trauma or facial plastic surgery. Their mean age was 25.6 (range, 19–35) years. The nasal base was measured and analyzed individually.

The surgical group comprised 54 patients with poor nasal base morphology who desired surgical improvement. They had undergone nasal-tip plasty from July 2009 to August 2013. All women in the surgical group were tested by Angelpsychology evaluation system (Bit Science & Technology Co. Ltd., Guangzhou, China). They were psychologically and physiologically healthy and had no history of nasal plastic surgery. Their mean age was 23.4 (range, 18–34) years. We measured the nasal base morphology preoperatively and >6 months postoperatively.

### Measurements

All patients underwent analysis using a computer-assisted system of measurement and analytical software for beauty on a ViewSonic computer (Bit Science & Technology Co. Ltd., Guangzhou, China). This system also included a video camera (G11; Canon, Tokyo, Japan).

With the patients seated 1.0 m away from the video camera, standard digital photographic images were taken in the basilar view. When in the basilar view, the nasal tip was connected with both sides of the cornea (Dongxue 2005). Using measurement and analysis software to take linear and angular measurements of the nasal base image, we obtained data for both the control group and surgical group. In the surgical group, these parameters were obtained before and after surgery.

### Standard linear landmarks

**Fig. 1:**
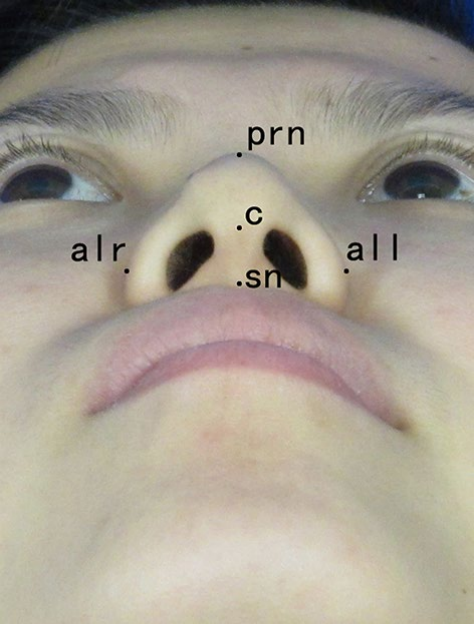
Nasal landmarks in the basilar view

The soft landmarks were the pronasale (prn), the most anterior midpoint of the nasal tip; the top point of the columella (c); the subnasale (sn), the midpoint of the columella base at the columella–labial junction; and the alare (alr, all), the most lateral point on each alar contour (He et al. 2009). The linear landmarks were the nasal base height (sn-prn), columella length (c-sn), and nasal width (alr-all) (Ghazipour et al. 2009) (Fig. 1).

### Standard angular landmarks

**Fig. 2:**
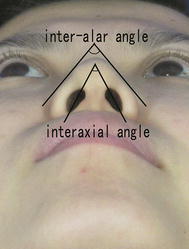
Inter-axial angle and inter-alar angle

For angular measurements, landmarks were placed at the upper and lower poles of the nostrils, through which the nostril axis runs. The upper pole of the nostril is located where the alare meets the upper end of the columella. The lower pole is located where the alare meets the nostril floor. The inter-axial angle is formed at the apex where the two nostril axes meet. The inter-alar angle is formed near the nose tip by the two tangents touching the alare on either side (Aung et al. 2000) (Fig. 2).

### Proportion indices related to the nasal base

**Fig. 3:**
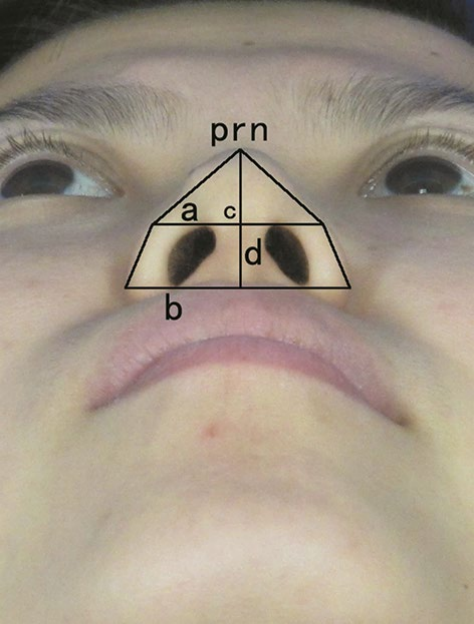
Nasal base partition

From the basilar view, we suggest that the nasal base includes both the upper part (containing the nasal tip triangle) and the lower part (containing the nostril trapezoidal) (Yang et al. 2012). Hence, we suppose that the upper bottom (a) connects both sides of the upper poles of the nostril intersection at the alar rim tangent, which acts as the bottom of the nasal tip triangle (i.e., the upper bottom part of the nostril trapezoidal). Additionally, the lower bottom (b) connects both sides of the lower poles of the nostril intersection at the alar rim tangent, which acts as the lower bottom of the nostril trapezoidal. The height of the nasal tip triangle is (c-prn). The height of the nostril trapezoidal is the columella length (c-sn) (Fig. 3).

We hypothesized that the nasal width (all-alr) was the median line of the trapezoid. We used the proportion index of height and bottom edge in the nasal tip triangle (i.e., c-prn/a) as well as the proportion index of the height and median line in the nostril trapezoidal (i.e., c-sn/all-alr) to embody the shape of the nasal tip portion and nostril portion. Farkas et al. (1986, 1994) studied the proportion indices of the nasal soft tissue in detail. Based on their proportion indices, we chose the nasal base protrusion-width index (sn-prn/alr-all). Thus, we made three linear measurements, two angular measurements, and three proportion indices, thereby making a total of eight indices.

### Surgical procedure

The surgical methods implemented for adjusting the nasal tip include suturing, excision, and transplantation (Mao et al. 2008; Toriumi 2006; Jang et al. 2007). All plastic surgery procedures were performed by Dr. Tan. For the surgical group, we used a transdomal suture for intermediate crural and lateral crural cephalic partial truncation (Beaty et al. 2002), autogenous cartilage transplantation as a columellar strut, and an onlay graft in the nasal tip (Bottini et al. 2008).

#### Transdomal suture

The bilateral intermediate dome underwent horizontal mattress-type suturing.

#### Lateral crural partial truncation

If the alar cartilage is well developed, we can resect no more than 40 % of the lateral crura in a lateral-to-medial direction (Beaty et al. 2002).

#### Columellar strut

When transplanting the partial septal cartilage to the medial crura as a columellar strut, the top is below the dome.

#### Onlay graft

The remaining septal cartilage is trimmed to a round shape with a diameter of about 3–5 mm; overlap is created in three to four layers and fixed on the new intermediate dome (Bottini et al. 2008).

### Statistical analysis

Data were analyzed using SPSS version 21 (IBM Corp., Armonk, NY, USA). An independent-sample *t*-test was used when data in the control group were compared with preoperative and postoperative data in the surgical group. The paired-samples *t*-test was used for comparison of preoperative and postoperative data in the surgical group. A 5 % level of significance (*P* < 0.05) was used for all analyses.

## Results

Linear measurements were in centimeters, and angular measurements were in degrees. Data regarding linear measurements, angular measurements, and proportion indices are shown in Tables 1, 2, 3 and 4.

**Table 1:** Comparison between control group (n = 101) and foreign reports

Index	Control	Oriental females (Aung et al. 2000)	Chinese female (Farkas et al. 1994)	Caucasian female (Farkas 1994)
	$\bar{x} \pm s$	$\bar{x} \pm s$	$\bar{x} \pm s$	$\bar{x} \pm s$
1 sn-prn	1.93 ± 0.20	1.67 ± 0.20	1.54 ± 0.18	1.93 ± 0.19
2 c-sn	0.90 ± 0.13			
3 all-alr	3.74 ± 0.37	3.76 ± 0.35	3.72 ± 0.21	3.14 ± 0.19
4 inter-alar angle	89.66 ± 8.53	90.89 ± 12.55		
5 inter-axial angle	78.09 ± 11.39	80.67 ± 10.96		
6 sn-prn/all-alr	0.52 ± 0.07	0.45 ± 0.06		0.61
7 c-prn/a	0.33 ± 0.05			
8 c-sn/all-alr	0.25 ± 0.04

**Table 2:** Comparison between control group (n = 101) and preoperative parameters in surgical group (n = 54)

Index	Control	Preoperative	t	*P*
	$\bar{x} \pm s$	$\bar{x} \pm s$		
1 sn-prn	1.93 ± 0.20	1.84 ± 0.22	2.473	0.014*
2 c-sn	0.90 ± 0.13	0.84 ± 0.15	2.728	0.007*
3 all-alr	3.74 ± 0.37	3.62 ± 0.26	2.026	0.044*
4 inter-alar angle	89.66 ± 8.53	91.22 ± 11.18	−0.896	0.373
5 inter-axial angle	78.09 ± 11.39	74.99 ± 13.85	1.493	0.137
6 sn-prn/all-alr	0.52 ± 0.07	0.51 ± 0.55	0.926	0.356
7 c-prn/a	0.33 ± 0.05	0.32 ± 0.04	0.253	0.800
8 c-sn/all-alr	0.25 ± 0.04	0.23 ± 0.04	3.102	0.002*

*P < 0.05

Line unit: centimeter, Angle unit: degree

**Table 3:** Comparison between control group (n = 101) and postoperative parameters in surgical group (n = 54)

Index	Control group	Postoperative	t	*P*
	$\bar{x} \pm s$	$\bar{x} \pm s$		
1 sn-prn	1.93 ± 0.20	2.10 ± 0.22	−4.785	0.000*
2 c-sn	0.90 ± 0.13	0.93 ± 0.13	−1.207	0.229
3 all-alr	3.74 ± 0.37	3.58 ± 0.36	2.627	0.009*
4 inter-alar angle	89.66 ± 8.53	80.93 ± 8.01	6.194	0.000*
5 inter-axial angle	78.09 ± 11.39	67.66 ± 12.69	5.217	0.000*
6 sn-prn/all-alr	0.52 ± 0.07	0.59 ± 0.42	−6.729	0.000*
7 c-prn/a	0.33 ± 0.05	0.4 ± 0.04	−9.896	0.000*
8 c-sn/all-alr	0.25 ± 0.05	0.26 ± 0.05	−2.495	0.014*

*P < 0.05

Line unit: centimeter, Angle unit: degree

**Table 4:** Comparison of preoperative and postoperative parameters in surgical group (n = 54)

Index	Preoperative	Postoperative	Difference	t	*P*
	$\bar{x} \pm s$	$\bar{x} \pm s$	$\bar{x} \pm s$		
1 sn-prn	1.84 ± 0.22	2.10 ± 0.22	−0.26 ± 0.27	−7.04	0.000*
2 c-sn	0.84 ± 0.15	0.93 ± 0.13	−0.09 ± 0.11	−6.05	0.000*
3 all-alr	3.63 ± 0.26	3.58 ± 0.36	0.05 ± 0.29	1.22	0.229
4 inter-alar angle	91.22 ± 11.18	80.93 ± 8.01	10.28 ± 8.58	8.81	0.000*
5 inter-axial angle	74.99 ± 13.85	67.66 ± 12.69	7.33 ± 8.99	5.99	0.000*
6 sn-prn/all-alr	0.51 ± 0.55	0.59 ± 0.42	−0.08 ± 0.05	−11.65	0.000*
7 c-prn/a	0.32 ± 0.04	0.4 ± 0.04	−0.08 ± 0.05	−11.589	0.000*
8 c-sn/all-alr	0.23 ± 0.04	0.26 ± 0.05	−0.04 ± 0.04	−7.711	0.000*

*P < 0.05

Line unit: centimeter, Angle unit: degree

In Table 1, we advised the proportion index of the nasal tip triangle (i.e., c-prn/a = 0.33 ± 0.05 ≈ 1:3) as well as the proportion index of the nostril trapezoidal (i.e., c-sn/all-alr = 0.25 ± 0.04 ≈ 1:4). Each value shows the shape of the nasal tip portion and nostril portion, respectively. Larger proportion indices indicate greater height and a narrower bottom; therefore, the corresponding portion morphology is higher. Conversely, smaller indices indicate lower height and a broader bottom; therefore, the corresponding portion morphology is flatter.

As shown in Table 2, indices 1 (sn-prn), 2 (c-sn), 3 (all-alr), and 8 (c-sn/all-alr) were significantly different. Compared with the control group, preoperative data in the surgical group showed the deformities: a lower nasal base height and a narrower nasal width. Although the difference in the nasal base protrusion-width was not statistically significant, the nostril portion morphology was still flatter based on the nostril proportion index.

As shown in Table 3, indices 1 (sn-prn), 3 (all-alr), 4 (inter-alar angle) and 5 (inter-axial angle), 6 (sn-prn/all-alr), 7 (c-prn/a), and 8 (c-sn/all-alr) were significantly different. Compared with the control group, postoperative data in the surgical group showed a higher nasal base and narrower width, a smaller inter-alar angle and inter-axial angle, and a larger nasal base protrusion-width index; namely, the whole nasal base was higher. Furthermore, the nasal tip and nostril proportion indices were larger, which meant the nasal tip and nostril portion morphology were both higher.

As shown in Table 4, the indices 1 (sn-prn), 2 (c-sn), 4 (inter-alar angle), 5 (inter-axial angle), 6 (sn-prn/all-alr), 7 (c-prn/a), and 8 (c-sn/all-alr) were significantly different. Through comparison of preoperative and postoperative data in the surgical group, we found that the nasal base height increased after surgery, the inter-alar angle and inter-axial angle decreased, and the nasal base protrusion-width index simultaneously increased. Moreover, we found that the nasal tip and nostril portion morphology were higher than those seen preoperatively.

**Fig. 4:**
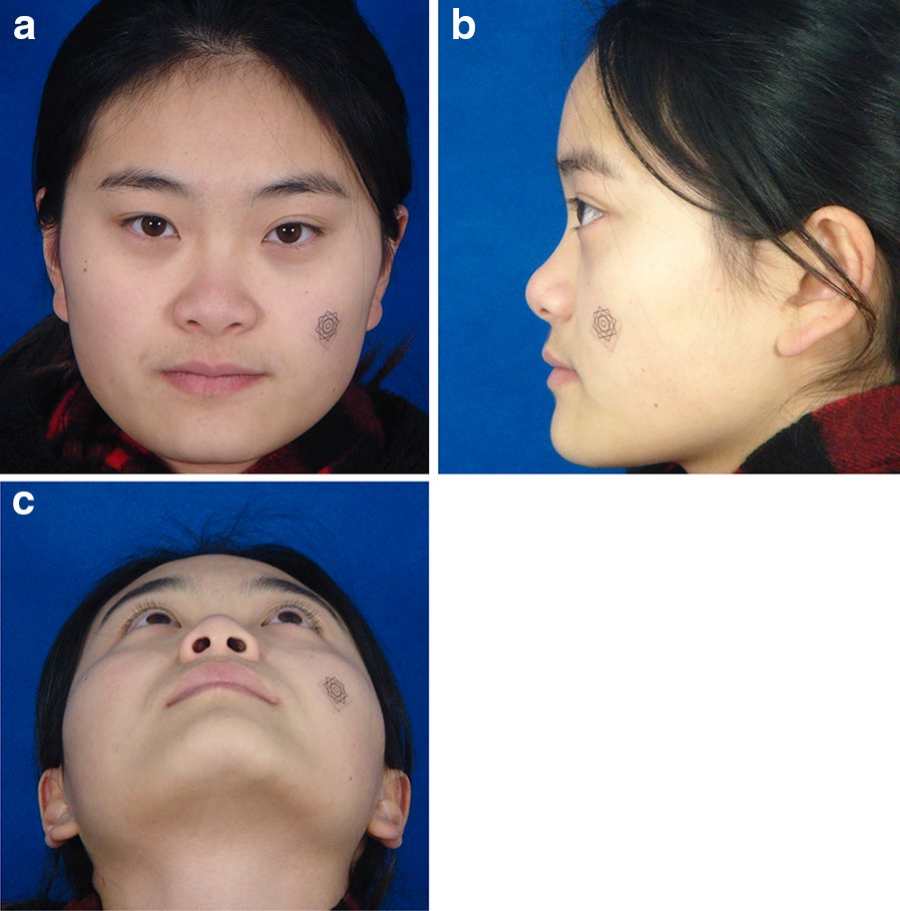
Preoperative photograph (**a** positive view, **b** lateral view, **c** basilar view)

**Fig. 5:**
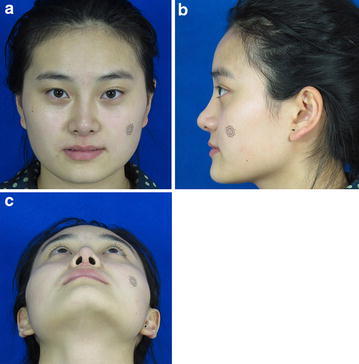
Photograph obtained 6 months postoperatively (**a** positive view, **b** lateral view, **c** basilar view)

**Fig. 6:**
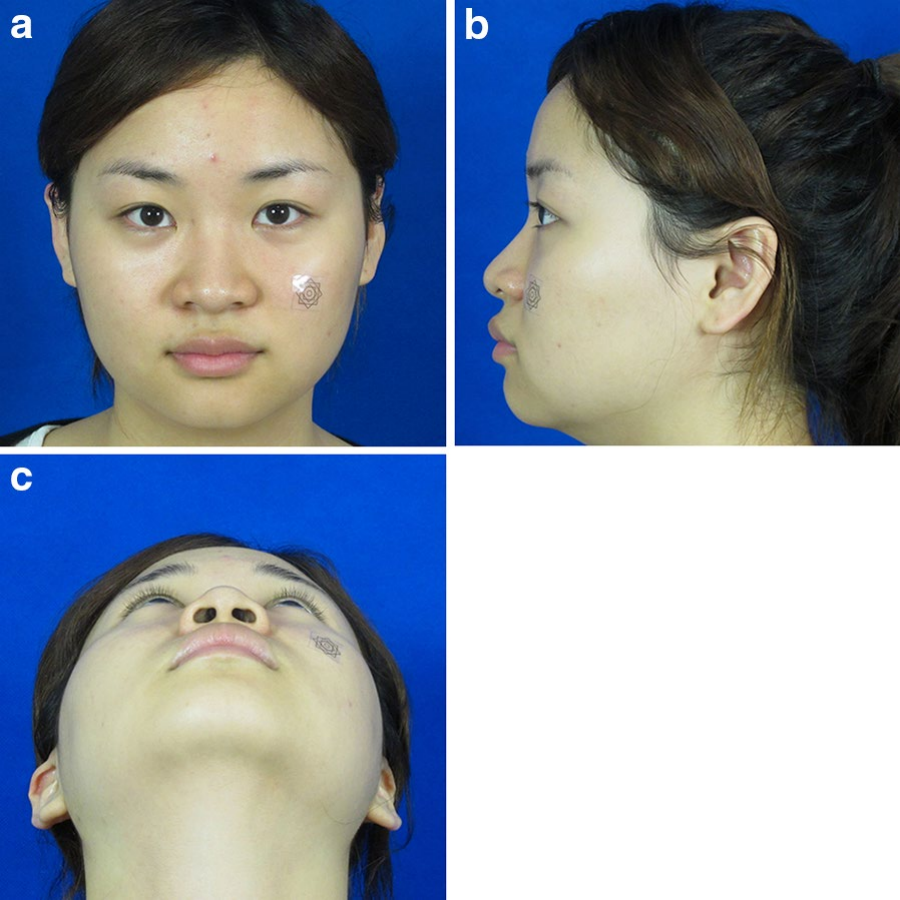
Preoperative photograph (**a** positive view, **b** lateral view, **c** basilar view)

**Fig. 7:**
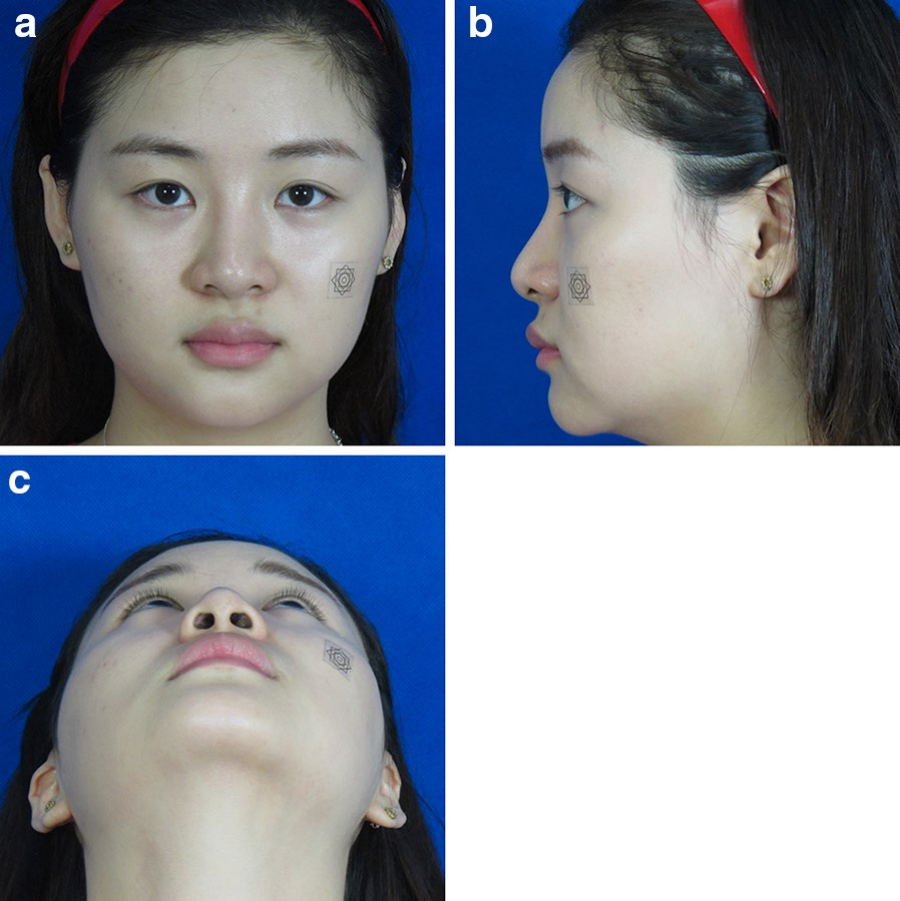
Photograph obtained 10 months postoperatively (**a** positive view, **b** lateral view, **c** basilar view)

All patients in the postoperative surgical group were followed up for 6–12 months (Figs. 4, 5, 6, 7). The postoperative photographs showed that the restrictive nasal tip and flared nostril were improved in all but three patients (5.56 %) who had only undergone graft deflection and needed secondary adjustment. We found no instances of other complications, such as infection, hematoma, perforation, or absorption of grafted cartilage. Moreover, no adverse functional effects were observed.

## Discussion

Clinical application of study data has shown that the nasal morphology has specific characteristics among different races and ethnicities (He et al. 2009; Wang et al. 2009; Ofodile and Bokhari 1995; Ofodile et al. 1993; Aung et al. 2000; Farkas 1994; Farkas et al. 1986; Li et al. 2014). These morphological particularities must be considered when planning a rhinoplasty. Much fewer data from non-Western than Western countries are available in this research field.

To better appreciate the significance of the linear measurements and how they relate to the nasal morphology, nasal indices should provide a better understanding of the proportion and ratio of different parts of the nose (Aung et al. 2000). Too many linear and angular measurements of the nasal base may confuse surgeons; proportion indices could integrate the linear and angular aspects of the nose to better illustrate the morphology. The nasal base protrusion-width index described by Farkas (1994) and Farkas et al. (1986) shows the whole nasal base morphology. We choose specific proportion indices to reflect the morphology of the corresponding nasal base components.

In this study, we performed and analyzed measurements of 155 Chinese Han women. In the control group (101 women), we measured the nasal base height, nasal width, and apex angles. Based on these data, we calculated the following proportion indices: (sn-prn/all-alr), (c-prn/a), and (c-sn/all-alr).

In this study, the inter-alar angle was 89.66° ± 8.53° and the inter-axial angle was 78.09° ± 11.39°. These values are close to the 90.89° ± 12.50° and 80.67° ± 10.96°, respectively, reported by Aung et al. (2000) in Oriental females. The protrusion-width index (sn-prn/all-alr) was 0.52 ± 0.07. This value is close to that reported by Sheng-han et al. (2010) in Chinese females (0.51 ± 0.08) and close to that reported by Li et al. (2014) in Han females (0.5456 ± 9.7). In a comparison with foreign reports (Table 1), we also found that the nasal width was wider and the protrusion-width index (sn-prn/all-alr) was significantly smaller than in Caucasian females in previous control groups. This conforms to the general view of aesthetic differences between Easterners and Westerners. The protrusion-width index (sn-prn/all-alr) of Caucasian females in the study by Farkas (1994) was 0.61, which is closer to the golden ratio (0.618). The control group represents the general morphology of Chinese Han women’s nasal base as characterized by flared nostrils and a restrictive nasal tip. The “typical” Asian nose is characterized by a bulbous appearance, flared nostrils, and nasal tip restriction (Mao et al. 2008). The nasal shape is not as stereoscopic as that of Westerners.

We chose classic surgical methods with which to contour the nasal base, which is well embodied in the corresponding proportion indices. In the surgical group, the change in the nasal base shape between the preoperative and postoperative period is related to the change in the supporting structure. The domal stabilization suture provides a method with which to unify, align, and stabilize the individual domal segments (Corrado et al. 2009). Transdomal suturing decreases the width of the dome arch and provides a small amount of projection from each side dome (Wang et al. 2009). It decreases the domal distance, narrows the nasal tip, strengthens the upward support, and increases the tip support. Autogenous cartilages are transplanted to the tip as onlay grafts create a more projecting and natural tip contour. This increases the nasal tip projection. Autogenous cartilage is transplanted between the medial crura and sutured together as a columellar strut, which adds the columellar length and provides support strength along the columella (Wang et al. 2009). Addition of medial crural support along the struts is commonly applied to increase tip projection, enhance tip strength, and correct buckling of the intermediate and medial crura (Kal et al. 2008).

Therefore, we found that the nasal base height and protrusion-width index increased, the inter-alar angle and inter-axial angle decreased in the postoperative surgical group. In terms of the new proportion indices of the nasal base, the domal stabilization suture and onlay graft increased the index (c-prn) (Sheng-han et al. 2010); this increased the proportion index of the nasal tip triangle. Autogenous cartilage transplantation as a columellar strut increases the height of the nostril; meanwhile, the alar base remains unchanged and the nasal width changes little, so the proportion index of the nostril trapezoidal also increases.

Comparing the surgical group with the control group and summarizing the changes in the new nasal base proportion indices, we found that the nostril part morphology in the preoperative surgical group was flatter than that in the control group. After surgery, the nasal tip and nostril part morphologies were higher than those in the control group. Changes in the new proportion indices meant that the flared nostrils and the restrictive nasal tip were improved, and the nasal base morphology became stereoscopic.

The subunit proportion indices intuitively reflect the nasal base morphology. When carrying out aesthetic surgery of the Chinese Han female nose, knowledge of the corresponding proportion indices will substantially contribute to surgical planning and subsequent postoperative assessment of outcomes.

The nasal base data obtained herein can also be used as reference standard in cleft lip repair, nasolabial deformity reconstruction, and nasal trauma repair. During the operation, the surgeon must pay attention to the characteristics of the external nose, avoid excessive correction, and maintain facial harmony.

There are no relevant reports about the nasal base proportion indices (c-prn/a) and (c-sn/all-alr), and there is no evaluation standard. This study on Han Chinese women has provided relevant data on the lower part of the nasal subunit and a morphological basis for nasal aesthetic evaluation and operative design. More randomized studies and comparative studies involving Han men are needed to establish aesthetic standards of the external nasal base.

## Conclusion

The data in this study provide a credible and objective reference for soft tissue evaluation of the external nasal base and surgical planning. These results can also serve as a useful guide for preoperative and postoperative evaluations of nasal base deformities. The proportion indices related to the nasal base are easy to obtain, and they clearly illustrate the nasal base morphology.
